# Mechanical Thrombectomy for Acute Ischemic Stroke in Patients With Cardiac Myxoma: A Case Series and Pooled Analysis

**DOI:** 10.3389/fneur.2022.877056

**Published:** 2022-04-18

**Authors:** Jie Rao, Zi Tao, Qiongqiong Bao, Mengbei Xu, Mingxia Jiang, Xiongpeng Weng, Bo Yin, Dandong Li, Yan Li, Xueli Cai, Fangwang Fu

**Affiliations:** ^1^Department of Neurology, The Second Affiliated Hospital and Yuying Children's Hospital of Wenzhou Medical University, Wenzhou, China; ^2^Department of Neurology, The Fifth Affiliated Hospital of Wenzhou Medical University, Lishui, China; ^3^Department of Neurology, Affiliated Yueqing Hospital, Wenzhou Medical University, Wenzhou, China; ^4^Department of Neurology, Ningbo Second Hospital, Ningbo, China; ^5^Department of Rehabilitation, The First Affiliated Hospital of Wenzhou Medical University, Wenzhou, China; ^6^Department of Neurology, Huangyan Hospital, Wenzhou Medical University, Taizhou, China; ^7^Department of Neurosurgery, The Second Affiliated Hospital and Yuying Children's Hospital of Wenzhou Medical University, Wenzhou, China

**Keywords:** thrombectomy, stroke, myxoma, large vessel occlusion, intravenous thrombolysis, endovascular therapy

## Abstract

**Background and Purpose:**

Acute ischemic stroke (AIS) is a common and life-threatening complication of patients with cardiac myxoma (CM). The role of the mechanical thrombectomy (MT) technique in CM-AIS patients remains unclear, and no guidelines exist for this population. Therefore, we conducted a case series study of MT in CM-AIS patients to investigate its safety and efficacy via a pooled analysis of published literature.

**Methods:**

Eleven CM-AIS patients who underwent MT between 2016 and 2021 were screened from multicenter stroke databases. Clinical, procedural, and outcome data were obtained from medical records. A systematic review was conducted to identify additional cases from published studies by searching PubMed and China National Knowledge Infrastructure databases. We then performed a pooled analysis of the published cases.

**Results:**

In the case series study, most patients were male (81.8%), with a median age of 51 years. All patients had CM located in the left atrium. The rate of successful reperfusion using the first-line thrombectomy technique was 100% with stent retriever (SR) and 66.7% with direct aspiration (DA), which resulted in overall successful reperfusion in 94.1% of all occlusions. The retrieved emboli of the five patients who underwent histopathology examination were identified as myxoma components. Hemorrhagic transformation was observed in five (45.5%) patients, of whom one was symptomatic (9.1%). Three-month favorable functional outcomes were achieved in five (45.5%) patients with a 3-month mortality rate of 18.2%. For the literature review, 35 cases with 51 target vessel occlusions were identified and included in the pooled analysis. The rate of successful reperfusion following first-line thrombectomy did not differ between SR (30 patients, 90.9%) and DA (10 patients, 83.3%). The overall successful reperfusion rate was 91.8% of all occlusions. Three-month favorable functional outcomes were achieved in 21 (60.0%) patients, and the mortality rate was 8.6%.

**Conclusions:**

Our findings suggest that MT is not only an effective technique but also a safe option for CM-AIS patients with large vessel occlusion. MT has several advantages for this population, which include a high recanalization rate, low bleeding risk, and the ability to evaluate the source of emboli and the etiology of stroke.

## Introduction

The occurrence of an embolic event is common in patients with cardiac myxoma (CM) and can contribute to a poor prognosis (1). Embolic events occur in 30–50% of CM patients, of which more than half occur in the cerebrovascular system (2, 3). CM-related ischemic stroke is the most common event, with an estimated incidence ranging from 10 to 30% of CM patients, albeit accounting for only 0.5% of all strokes (3, 4). Because of the rarity of CM-related stroke, prospective studies and clinical randomized controlled trials are not possible. To date, only a few studies have provided evidence for effective therapeutic strategies for CM-related stroke, most of which derive from case reports or small case series. Intravenous thrombolysis (IVT) treatment administered within 4.5 h of the stroke remains the mainstay of reperfusion treatment for most acute ischemic stroke (AIS) patients (5). However, evidence on the efficacy and safety of IVT in CM-related AIS (CM-AIS) are conflicting. The incidence of intracranial hemorrhage (ICH) and parenchymal hematoma (PH) type II are 22.7 and 18.2% in patients with CM-AIS who receive IVT (6). IVT carries a higher risk of hemorrhage in patients with CM-AIS than in those with strokes of other causes. Thus, IVT may be reasonable in only select patients whose potential benefit from IVT outweighs the risk of hemorrhage. Currently, mechanical thrombectomy (MT) using stent retrievers (SRs) or direct aspiration (DA) is recommended as first-line treatment for AIS patients with large vessel occlusion (LVO). Moreover, MT has been demonstrated to be safe and effective in patients with contraindications for IVT, which include previous anticoagulant treatment and a history of ischemic or hemorrhagic stroke, intracranial aneurysms, or concomitant intracerebral hemorrhage (7). Although patients with CM-AIS often present with LVO, few case reports have addressed the application of MT in patients with CM-AIS. Thus, there is a need to evaluate the efficacy and safety of MT in patients with CM-AIS.

Considering the clinical uncertainties related to reperfusion treatment in patients with CM-AIS, the present study aimed to elucidate the clinical features, safety, efficacy, and outcomes of MT in patients with CM-AIS. This is the largest case series reporting the use of MT in patients with CM-AIS. Additionally, we reviewed all previous case studies of patients who received MT.

## Materials and Methods

### Study Design

This study consisted of two parts: the first part reported 11 cases from our multicenter databases. The second part comprised a comprehensive literature review. This study was approved by the local institutional review board. The study was a retrospective review of electronic medical records, and informed consent for publication was obtained from all patients and their families.

### Retrospective Multicenter Case Series

#### Stroke Management Protocol

A standard protocol for the evaluation and treatment of patients with AIS with LVO was established and implemented in all participating centers according to the 2019 Chinese Stroke Association guidelines for clinical management of ischemic stroke (5) as below:

(1) All patients presenting with stroke symptoms were clinically assessed by a multidisciplinary stroke team on admission. The National Institute of Health Stroke Score (NIHSS) and modified Rankin Scale (mRS) score were immediately assessed by stroke neurologists. Meanwhile, a stroke nurse placed peripheral venous catheters and obtained blood samples for emergency laboratory examination. A non-contrast computed tomography (CT) of the brain and an Alberta Stroke Program Early CT Score (ASPECTS) was obtained as soon as possible. CT angiography (CTA) and/or CT perfusion were performed depending on NIHSS score and onset-to-door time. Magnetic resonance imaging (MRI) was considered an alternative to CT in some patients.
(2) IVT treatment with alteplase was started prior to MT as soon as possible if patients fulfilled the criteria for IVT within 4.5 h of stroke onset. The efficacy of IVT was evaluated by stroke neurologists and sent immediately to the neurointerventional department. MT was started immediately if the patient was ineligible for IVT. The reperfusion treatment strategy, which included thrombectomy techniques and devices, was determined according to the discretion of the stroke neurologists and neurointerventionalists. Successful revascularization was defined as a thrombolysis in cerebral infarction grading scale (TICI) score of 2b/3. If the first-line device failed to achieve successful recanalization, rescue therapy was performed, which included angioplasty, stent implanting, and the use of other thrombectomy devices. If conditions permitted, retrieved emboli were obtained and sent for histopathological examination.
(3) All patients were clinically assessed by stroke neurologists 24 h after MT. Follow-up CT or MRI was performed at 24 ± 12 h after MT and repeated as necessary. The presence of hemorrhagic transformation (HT) and symptomatic ICH was ascertained in post-MT imaging data according to the criteria of the European Cooperative Acute Stroke Study II (ECASS-II) (8).
(4) The etiology, diagnosis, and risk factor assessments of AIS were initiated after MT. Blood lipid, glucose metabolism, serum homocysteine, and carotid ultrasound tests were performed to identify atherosclerosis. Transthoracic echocardiography (TTE) and Holter monitoring were routinely performed as part of the diagnostic work-up for cardioembolic stroke. Transesophageal echocardiography (TEE) was performed in patients who were highly suspected of having a cardioembolic stroke if TTE failed to detect the source of the cardioembolic stroke. Each patient was classified into a specific etiological category according to the Trial of ORG 10172 in Acute Stroke Treatment categories. CM-AIS was considered as the stoke etiology if one or more of the following criteria were met: (1) The retrieved embolus was identified as a part of myxoma; (2) cardioembolic stroke in CM patients without other common cardioembolic etiologies, including atrial fibrillation and mitral stenosis; (3) if the cardioembolic stroke occurred in CM patients with other potential stroke etiologies, the appearance of the embolus should differ from that of commonly observed thrombi (e.g., jelly-like, gelatinous, and fatty appearance).
(5) Patients were assessed by stroke neurologists using the mRS 3 months after stroke onset, either by telephone or outpatient visit. In patients who were lost to follow-up, the mRS score at discharge was used instead. An mRS score change of ≤2 between premorbid and 3 months was defined as a favorable functional outcome. Safety outcomes included the development of ICH or symptomatic ICH (sICH) and 3-month all-cause death. The recurrence of embolic events was also recorded at the 3-month follow-up.

#### Patient Selection

We retrospectively reviewed all consecutive AIS patients who underwent MT between January 2016 and December 2021 from our stroke registry database, which is a multicenter retrospective collaboration of seven comprehensive stroke centers across Zhejiang, Southeastern China. These registries included all AIS patients who were admitted within 7 days of providing proof of good health. All consecutive patients who fulfilled the following criteria were included: (1) aged 18 years or older; (2) diagnosis of AIS with LVO confirmed by digital subtraction angiography (DSA); (3) treated with MT within 24 h of symptom onset; (4) diagnosis of CM confirmed via cardiac pathological examination or echocardiography; (5) fulfilled the criteria of CM-AIS mentioned above. Patients were excluded if they met the following criteria: (1) treated with intra-arterial thrombolysis alone; (2) a pre-stroke mRS score > 2; (3) presence of severe cerebral artery stenosis; (4) other stroke etiologies, such as malignant tumor and Moyamoya disease.

#### Data Collection

All stroke-related data were obtained from the medical records system by two trained neurologists who were blinded to all clinical and outcome data. Data comprised demographic characteristics, risk factors for stroke, clinical data (i.e., clinical presentations, prior IVT, pre-stroke mRS score, and NIHSS score at admission and 24 ± 12 h after MT), laboratory findings, imaging data (ASPECTS score, occlusion site, collateral flow, and the presence of HT), procedural characteristics [i.e., thrombectomy techniques and devices used, the total number of the device passes, degree of recanalization, time of onset to recanalization or final angiography (OTR), procedural time (groin puncture to recanalization time), and procedural complications], and outcome data (i.e., sICH, recurrence of embolism, 3-month mRS score, and mortality).

The location, tumor surface, and maximum tumor diameter of CM were obtained from the ultrasound or pathology database. Possible clinical manifestations of CM were screened using medical records. Surgery information, surgical complications, and the utilization of antithrombotic therapy during the interval between AIS onset and CM surgery were reviewed if available. Histopathological characteristics of CM and the retrieved embolus, if available, were obtained from the medical records system.

### Systematic Review

**Figure 1:**
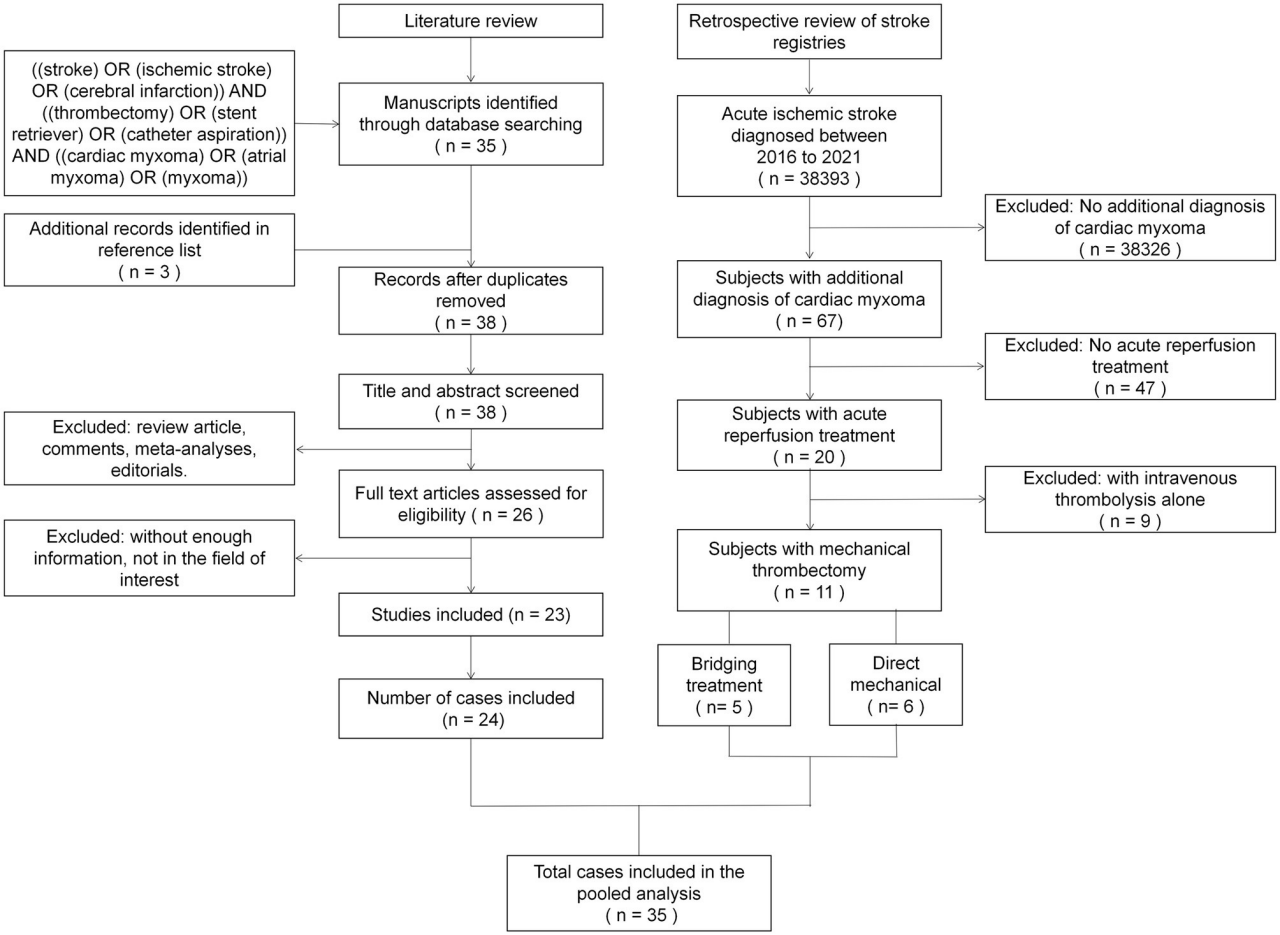
Literature review and retrospective review flow charts.

We performed a systematic review of the published literature by searching PubMed and China National Knowledge Infrastructure (CNKI) databases according to the Preferred Reporting Items for Systematic Reviews and Meta-analyses guidelines on December 30, 2021 (a flow diagram of the procedure is shown in Figure 1). The search strategy was: (“stroke” OR “ischemic stroke” OR “cerebral infarction”) AND (“thrombectomy” OR “stent retriever” OR “catheter aspiration”) AND (“cardiac myxoma” OR “atrial myxoma” OR “myxoma”). Reference lists of identified publications were manually searched to find relevant publications that were not captured by the initial search strategy. No restrictions on language or date of publication were applied. All types of articles were eligible for inclusion, including case reports and case series. Two authors independently screened all included studies, and a predefined standardized data collection form was used to collect relevant information from each eligible article.

Data from our cases series and systematic review were analyzed separately. Descriptive statistics are presented as means (standard deviations), numbers (proportions), medians [interquartile ranges (IQRs)], or medians (ranges). Formal statistical comparisons were not performed because of the small sample sizes.

## Results

### Retrospective Multicenter Case Series

#### Patient and Clinical Characteristics

**Figure 2:**
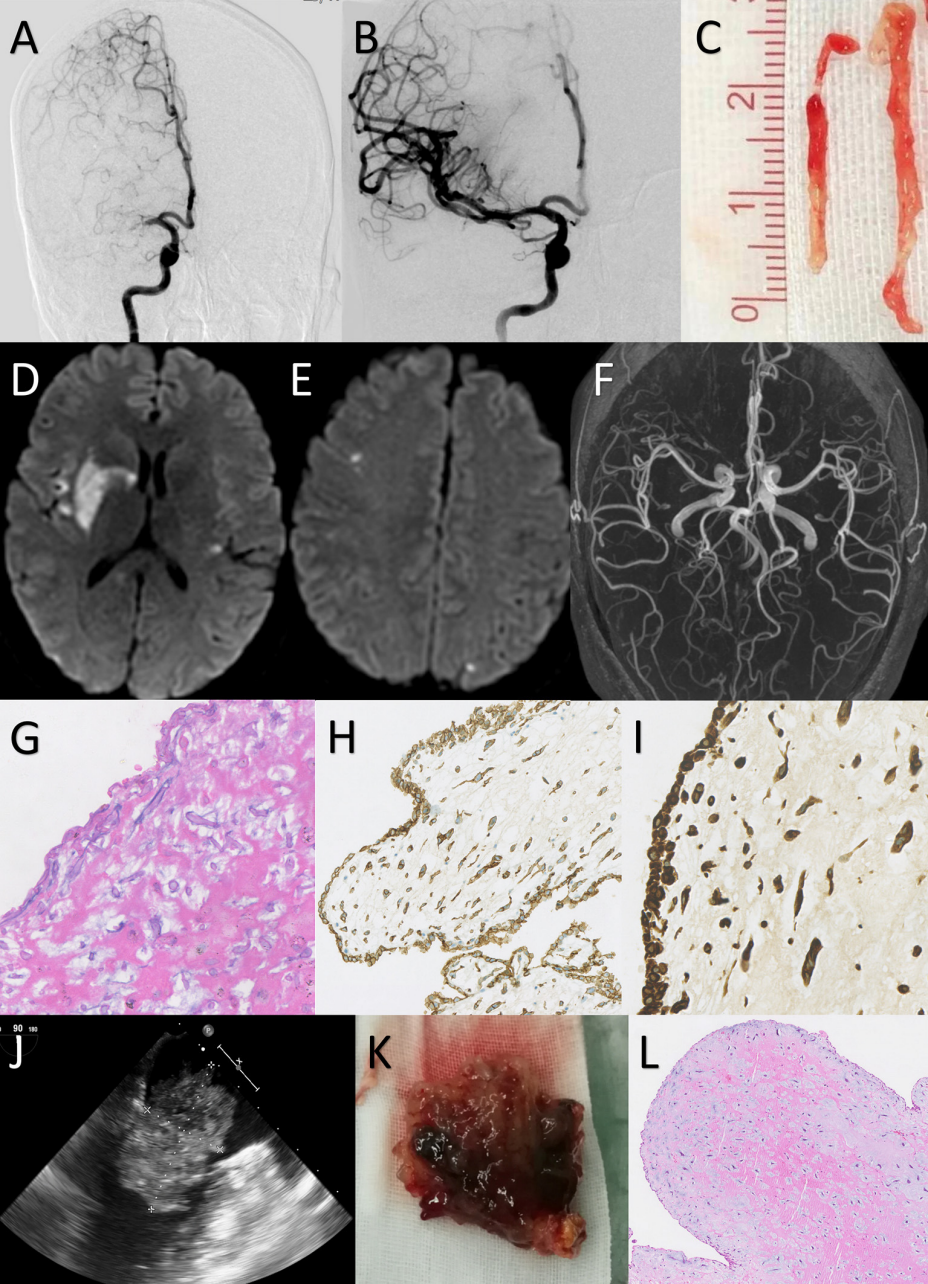
Case 1: a 38-year-old female presented with an NIHSS score of 11 points. Mechanical thrombectomy was performed due to the absence of early neurological improvement with intravenous thrombolysis. **(A)** Digital subtraction angiography confirmed that the right M1 segment of the middle cerebral artery (MCA) was occluded. **(B)** After the first attempt with Trevor 4 × 20 mm stent retriever, angiography showed complete recanalization of the MCA with a TICI score of 3. **(C)** The gross appearance of retrieved embolus: long yellowish and gelatinous tissue. **(D,E)** The diffusion-weighted magnetic resonance imaging on Day 3 showed acute infarction in the right basal ganglia and bilateral cerebral cortex. **(F)** Magnetic resonance angiography on Day 3 showed no vascular abnormalities. **(G–I)** Hematoxylin and eosin (H&E) staining of the retrieved embolus demonstrates round and spindle cells with a myxoid matrix. Staining for CD34 and vimentin were strongly positive. This histopathological view indicated the embolus was originated from cardiac myxoma. [**G**: H&E staining 200×; **H**: CD34 staining 100×; **I**: vimentin staining 200×] **(J)** Transesophageal echocardiogram showed a 53 × 56 mm myxoma in the left atrium. **(K)** Gross examination revealed a 55 × 58 × 40-mm-sized reddish-brown myxoid mass with an irregular surface. **(L)** Histological examination of the removed cardiac mass showed myxoma cells singly within the myxoid matrix, confirming the diagnosis of a cardiac myxoma.

A total of 38,393 AIS patients were enrolled in the stroke registries during the study period. Of these, 67 patients (1.75‰) were identified as having CM-AIS. Twenty patients with CM-AIS were treated with reperfusion, of whom nine were treated with IVT alone, six with MT alone, and five with bridging therapy. As a result, we identified 11 CM-AIS patients treated with MT. The individual clinical details are described in Table 1, and the characteristics of the cohort are summarized in Table 2. A typical case of MT-treated CM-AIS was described in Figure 2. The median age was 51 years (range 38–83), and most patients were male (81.8% male). Four patients (36.4%) had traditional stroke risk factors, of which hypertension (three patients, 27.3%) was the most common. Patient 5 had type 2 diabetes, and Patient 7 had atrial fibrillation. Two patients developed AIS despite prior antiplatelet and anticoagulation treatments, respectively. The median NIHSS score at admission was 22 (IQR 12–26). None of the patients who underwent prior IVT showed a rapid improvement in neurological symptoms. The clinical condition of one patient deteriorated following IVT.

**Table 1:** Characteristics of CM-AIS patients who underwent mechanical thrombectomy.

	**Age (year)**	**Sex**	**Risk factors**	**Initial NIHSS**	**HDA**	**Multi-vessel involved**	**IVT (effect)**	**Mechanical thrombectomy**								**HT**	**sICH**	**Cardiac myxoma**				**Stroke relapse before removal**	**mRS at follow-up**
								**Occlusion site**	**Device, type, no. of pass, recanalization**	**TICI**	**Embolus (appearance, histology)**	**Complic-ation**	**OTR (min)**	**PTR (min)**	**NIHSS at 24 ±12 h**			**Imaging diagnostic method**	**Site and diameter**	**Symptom**	**Removal time**		
1	38	F	No	11	No	No	Yes (NE)	Right M1	Trevor Provue 4 × 20, SR, 1, pass	3	Gelatinous and elastic material. Myxoma without thrombus	No	248	59	0	No	No	TEE	Left atrium 51 mm	No	14 days	No	0
2	50	F	Hypertens-ion	22	No	No	Yes (NE)	Left ICA	Solitare FR 4 × 20, SR, 1, pass	3	Whitish-to-reddish, gelatinous embolus. Myxoma mixed with thrombus	No	243	53	20	No	No	TTE	Left atrium 48 mm	No	Not performed	No	4
3	51	M	No	25	No	Yes	Yes (NE)	Left M2	Intra-arterial thrombolysis *via* 5F catheter, 1, failed Headway27 microcatheter, DA, 2, failed	0	Gelatinous embolus. NA	No	247	36	19	Yes, PH2	No	TEE	Left atrium 41 mm	No	24 days	No	1
4	39	M	No	25	No	Yes	No	Right ICA BA Right VA	Trevo Provue 4 × 20, SR, 2, pass Trevo Provue 4 × 20, SR, 2, pass Trevo Provue 4 × 20, SR, 2, pass	3 2b 3	Gelatinous and friable embolus. Myxoma mixed with thrombus	Distal embolism	254	79	25	No	No	TTE	Left atrium 53 mm	No	Not Performed	No	6
5	52	M	Diabetes	12	No	No	Yes (NE)	Right M2	Trevo Provue 4 × 20, SR, 1, pass	2b	Fatty appearing embolus; NA	No	299	39	6	No	No	TTE	Left atrium 49 mm	No	17 days	Yes	0
6	83	M	Hypertens-ion	26	Yes	Yes	No	BA Right VA Right SA	5 Fr Navien distal access catheter, DA, 1, pass 8F guiding catheter, DA, 2, failed, then Appolo 2.5 × 13 mm intracranial stent, implantation, 1, pass Untreated	2b 3	Brown gelatinous material; NA	Tonsillar herniation	301	116	26	No	No	TTE	Left atrium 24 mm	Syncope Chest pain	Not performed	No	6
7	67	M	Hypertens-ion, atrial fibrillation	9	No	Yes	No	Right M2	Trevo Provue 4 × 20, SR, 4, pass	3	Fragile and gelatinous embolus; NA	Vasospasm	309	74	3	Yes, SAH	No	TTE	Left atrium 34 mm	Syncope	2 months	Yes	0
8	49	M	No	28	Yes	No	No	Left M1	Solitaire FR 6 × 20, SR, 1, pass	3	White and fragile embolus. Myxoma	Malignant infarction	271	73	10	Yes, PH2	No	TEE	Left atrium 44 mm	No	62 days	No	3
9	40	M	No	22	No	No	No	Left ICA Left M1	8F guiding catheter, DA, 4, pass Solitaire FR 6 × 20, SR, 2, pass	3 3	Gelatinous and white embolus; NA	Vasospasm	289	49	18	Yes, PH2	No	TTE	Left atrium 41 mm	No	3 months	No	3
10	52	M	No	18	No	No	No	Left M2	Solitaire FR 4 × 20, SR, 3, pass	3	Gelatinous material with thrombus; NA	No	196	25	16	No	No	TTE	Left atrium 60 mm	No	2 months	No	2
11	51	M	No	26	No	Yes	Yes (NE)	Left ICA Left A1 Left PCA	Trevo Provue 6 × 30, SR, 2, pass Trevo Provue 4 × 20, SR, 1, pass Trevo Provue 4 × 20, SR, 1, pass	3 3 2b	Fragile and gelatinous embolus. Myxoma	Distal embolism, malignant infarction	235	84	22	Yes, PH2	Yes	TTE	Left atrium 43 mm	No	50 days	No	5

*BA, basilar artery; CM-AIS, cardiac myxoma related acute ischemic stroke; DA, direct aspiration; F, female; HDA, high-density sign of artery; HT, hemorrhagic transformation; ICA, internal carotid artery; IVT, Intravenous thrombolysis; M, male; MCA, middle cerebral artery; mRS, modified Rankin Scale; NE, not effective; NIHSS, National Institutes of Health Stroke Scale; OTR, onset to recanalization time; PCA, posterior cerebral artery; PH2, parenchymal hematoma type II; PTR, puncture to recanalization time; SAH, subarachnoid hemorrhage; sICH, symptomatic intracranial hemorrhage; SR, stent retriever; TEE, transesophageal echocardiography; TICI, Thrombolysis in Cerebral Ischemia; TTE, transthoracic echocardiography; VA, vertebral artery*.

**Table 2:** Clinical characteristics, periprocedural and outcome results of CM-AIS patients.

	**Institutional cases (*n* = 11)**	**Literature cases (*n* = 24)**	**Total cases (*n* = 35)**
**Characteristics**
Median age, years (IQR)	51 (40–52)	42 (21–45.75)	42 (21–51)
Female sex, % (*n*)	18.2 (2)	54.5 (12)	40.0 (14)
Stroke risk factors, % (*n*)	36.4 (4)	12.5 (3)	20.0 (7)
Median inital NIHSS (IQR)	22 (12–26)	16 (11.5–24)	18.5 (12–25.75)
Median ASPECTS (IQR)	9 (8–10)	N.A.	N.A.
High density sign of artery, % (*n*)	18.2 (2)	9.5 (2)	12.9 (4)
Multivessel involvement, % (*n*)	45.5 (5)	45.8 (11)	45.7 (16)
Intravenous thrombolysis, % (*n*)	45.5 (5)	62.5 (15)	57.1 (20)
Cerebral aneurysms, % (*n*)	0	4.2 (1)	2.9 (1)
**Cardiac myxoma characteristics**
Prestroke clinical presentation, % (*n*)	18.2 (2)	16.7 (4)	17.1 (6)
Median tumor diameter, mm (IQR)	44 (41–51)	37.5 (25.5–47.5)	42 (34–51)
Tumor in left atrium, % (*n*)	100 (11)	95.8 (23)	97.1 (34)
Irregular tumor surface % (*n*)	63.6 (7)	N.A.	N.A.
Pre-removeal embolism, % (*n*)	18.2 (2)	8.3 (2)	11.4 (4)
**Side of occlusion, % (** ***n*** **)**
Anterior circulation	90.9 (10)	95.8 (23)	94.3 (33)
ICA	36.4 (4)	16.7 (4)	22.9 (8)
MCA M1	27.3 (3)	79.2(19)	62.9 (22)
MCA M2	36.4 (4)	12.5 (3)	20.0 (7)
ACA	9.1 (1)	8.3 (2)	8.6 (3)
Posterior circulation	27.3 (3)	16.7 (4)	20.0 (7)
BA	18.2 (2)	16.7 (4)	17.1 (6)
VA	18.2 (2)	4.2 (1)	8.6 (3)
PCA	9.1 (1)	8.3 (2)	8.6 (3)
PCoA	0	4.2 (1)	2.9 (1)
**Procedural characteristics**
Treated occlusions	17	34	51
Median total passages for occlusions (IQR)	2 (1–3)	1 (1–2)	1 (1–2)
Median total passages for patients (IQR)	3 (1–5)	1.5 (1–2.75)	2 (1–4)
First-line technique, % (*n*)			
Stent retriever	76.5 (13)	66.7 (20)	70.2 (33)
Direct aspiration	17.6 (3)	30.0 (9)	25.5 (12)
Intra-arterial thrombolysis	5.9 (1)	3.3 (1)	4.3 (2)
Successful recanalization with 1 pass	35.3 (6)	66.7 (20)	55.3 (26)
Successful recanalization with 1st device	88.2 (15)	84.4 (27)	89.4 (42)
Successful recanalization - final	94.1 (16)	90.6 (29)	91.8 (45)
Onset to recanalization, min (IQR)	254 (243–299)	N. A.	N. A.
Duration of intervention, min (IQR)	59 (39–79)	N. A.	N. A.
**Outcome**
Hemorrhagic transformation, % (*n*)	45.5 (5)	20.8(5)	28.6 (10)
Symptomatic ICH, % (*n*)	9.1 (1)	4.2 (1)	5.7 (2)
Decompressive craniectomy, % (*n*)	18.2 (2)	4.2 (1)	8.6 (3)
Mortality, % (*n*)	18.2 (2)	4.2 (1)	8.6 (3)
Median mRS (IQR)	3 (0–5)	2 (1–3)	2 (1–3)
Favorable function outcome, % (*n*)	45.5 (5)	66.7 (16)	60.0 (21)

*The percentages for subcategories are based on the patients who have related data*.

#### Imaging Characteristics

All patients had an ASPECTS of more than 8 points (median 9, range 8–10). A hyperdense artery sign was reported in 18.2% of patients. Using DSA, LVOs were identified in the anterior circulation alone for eight patients, the posterior circulation alone for one patient, and anterior and posterior circulations concurrently for two patients. Multivessel occlusions were identified in five cases. The most frequently involved vessel was the M2 segment of the middle cerebral artery (MCA) (four patients, 36.4%) and internal carotid artery (ICA) (four patients, 36.4%), followed by the M1 segment of the MCA (three patients, 27.3%), basilar artery (BA) (two patients, 18.2%), vertebral artery (VA) (two patients, 18.2%), posterior cerebral artery (PCA) (one patient, 9.1%), and anterior cerebral artery (ACA) (one patient, 9.1%). Thrombus migration was found in two patients. No cerebral aneurysms were found.

#### Myxoma Characteristics

AIS was the initial manifestation of CM in 81.8% of patients. Cardiac obstructive symptoms were reported as the initial manifestation in the remaining two patients and included syncope episodes in two patients and chest pain in one patient. CM was diagnosed after MT in the majority of the patients (10 patients, 90.9%). TTE was detected in 72.7% of the CMs, and TEE was detected in the remaining CMs in 27.3% of patients with a negative TTE. All CMs were located in the left atrium. The median diameter of CMs was 44 mm (range 24–60 mm). An irregular or villous surface was found in seven (63.7%) patients. Histological data of CMs were available in six patients, of whom three had thrombi that overlapped the myxoma. Malignant cells were not found in any patient.

#### Procedural Characteristics and Efficacy

The procedural details are outlined in Table 1. All patients received general anesthesia, and all endovascular procedures were performed *via* the standard femoral approach. The median OTR and procedure duration was 254 and 59 min, respectively. A total of 17 target vessel occlusions were treated as follows: four ICA, four M2, three M1, two BA, two VA, one ACA, and one PCA. Most target vessel occlusions were treated using first-line SR thrombectomy (13 patients, 76.5%). DA as the first-pass technique was applied to three occlusions (17.6%). First-attempt successful reperfusion was achieved in 38.5% occlusions with SR and 33.3% with DA. The rate of successful reperfusion using the first-line thrombectomy technique was 100% with SR and 66.7% with DA, which resulted in overall successful reperfusion in 94.1% of all occlusions and 90.9% of patients. Rescue treatments were administered to two patients. The median maneuver count was 2 (IQR 1–3) for all occlusions and 3 (IQR 1–5) for all patients. The median NIHSS score decreased from 22 to 18 (IQR 6–22) 24 h after MT. Transient vasospasm and distal embolism were recorded in two patients each. No cases of dissection were reported. The retrieved embolism fragments were usually gelatinous, elastic, and different from common thrombi. Five of these underwent histopathology examination and were later confirmed as myxoma components.

#### Post-procedural Treatment

After MT, five patients received antiplatelet treatment, and five patients received anticoagulant regimens; however, one patient did not receive any antithrombotic therapy because of the presence of an HT (Patient 11). Notably, neither prior anticoagulation nor antiplatelet therapy prevented stroke recurrence in one patient who received dual antiplatelet treatment and subsequent rivaroxaban, and another with low-molecular-weight heparin (LWMH). CM surgical excision was performed in eight (72.7%) patients. Three patients did not undergo surgery: two patients because of early death and one patient because of family refusal based on extremely poor general condition. The median time between AIS onset to surgical excision was 55 days (IQR 18.75–61.5). None of the patients presented with perioperative complications. After the surgery, CM recurrence or embolism events were not reported in any patient until the end of the study. The majority of patients received aspirin after the removal of the CM, and only one patient (Patient 7) was treated with rivaroxaban because of atrial fibrillation. Two (18.2%) patients underwent decompressive craniectomy because of a malignant infarction (Patients 8 and 11).

#### Outcomes

HT was identified in five cases (45.5%), of whom four had PHs (all PH2), and one had subarachnoid hemorrhage (SAH). Of these, only one (9.1%) had sICH with malignant brain edema, which required decompressive craniectomy. One patient died during hospitalization due to a cerebellar tonsillar hernia. The median mRS score at the 3-month follow-up was 3 (IQR 0–5). Favorable function outcomes at 90 days were achieved in five (45.5%) patients. The overall mortality rate at the 3-month follow-up was 18.2%.

### Cases in the Pooled Analysis

Our literature review rendered 22 case reports and one small case series. Twenty-four patients plus our 11 patients were included in the pooled analysis. Detailed results of the cases from the literature review are presented in Supplementary Table 1. All CMs were located in the left atrium (34 patients, 97.1%) except one, which was located in the left ventricle. It was noteworthy that only 27 (77.1%) patients were adults, which resulted in a median age of 42 years (IQR 21–51). Only five patients (14.3%) were aged over 60 years. There was a male predominance, whereby 60% of patients were male, and the median NIHSS score at admission was 18.5. IVT was performed in 20 patients (57.1%), of whom none showed rapid improvement in symptoms. LVOs were located in the anterior circulation in 33 patients (94.3%) and the posterior circulation in seven patients (20%). Multiple vessel involvement was reported in 16 (45.7%) patients. However, cerebral aneurysms were only found in one patient (2.9%). Thrombus migration was found in five (14.3%) patients. A total of 51 target vessel occlusions were recorded as follows: 23 M1, 10 M2, 7 ICA, 4 BA, 2 VA, 3 ACA, 1 PCA, and one posterior communicating artery (PCoA). Four of the occlusions did not have detailed information on the MT technique used. Most occlusions were treated with first-line SR thrombectomy (33/47 occlusions, 70.2%), 12 occlusions were treated with first-line DA thrombectomy, and the remaining two occlusions were treated with intra-arterial thrombolysis. First-attempt successful reperfusion was achieved in 53.1% (17/32 occlusions) of occlusions using SR and 75% (9/12 occlusions) of occlusions using DA. The rate of successful reperfusion with the first-line thrombectomy did not differ between SR (30/33 occlusions, 90.9%) and DA (10/12 occlusions, 83.3%). The median maneuver count was 1 (IQR 1–2) for all occlusions and 2 (IQR 1–4) for all patients. The overall rate of final successful reperfusion for all occlusions was 91.8% (45/49 occlusions). The retrieved thrombus was confirmed as CM tissue in 20 patients. The prevalence of HT and sICH was 28.6 and 5.7%, respectively. The prevalence of HT did not differ between patients who underwent IVT (five patients, 25%) and those who did not undergo IVT (five patients, 33.3%). Favorable functional outcomes were achieved in 21 (60%) patients. The total mortality rate was 8.6%.

## Discussion

Despite the rarity of CM-AIS, recognizing the efficacy and safety of reperfusion treatment for CM-AIS is crucial because of its high incidence and morbidity. To the best of our knowledge, this study is the largest case series of CM-AIS patients treated with MT. Moreover, this is the largest systematic review conducted on this topic. CM is the most common primary cardiac tumor and accounts for more than 50% of all cardiac tumors in patients aged over 16 years and nearly 21% of all cardiac tumors of patients aged younger than 16 years (9). The clinical manifestation of CM is diverse and varies from incidental detection on imaging in the asymptomatic population to sudden cardiac death (1–4). A triad of clinical symptoms has been recognized: valvular obstruction, constitutional symptoms, and embolization events (1–4). Among these, embolization events are reported in up to 50% of all CM patients (3). Cerebral embolization is relatively common and accounts for more than half of all embolic events (1, 4, 10). Although CM only accounts for <1% of AIS in the total population, if the common etiologies of AIS are not apparent, especially in young people and children, CM must be considered an important differential diagnosis. In most studies, a 2:1 female preponderance in the CM population has been reported (1, 2, 11). However, there was a male preponderance in both our case series and literature review. Similar to our findings, previous studies have reported smaller proportions of women than men among CM patients with ischemic stroke or embolic complications (ranging from 1 to 1.4:1) (12, 13). A possible reason for this phenomenon is that the traditional risk factor of cerebrovascular disease is more frequent among male patients. However, recent studies have shown that sex is not an independent risk factor for ischemic stroke and embolic events (14–16). Only tumor type, tumor size, surface irregularity are independently associated with the risk of embolism (16). Moreover, CHA_2_DS_2_-VASc scoring is recommended for predicting the risk of embolism in CM patients (15).

Regarding the clinical manifestations of CM in CM-AIS patients who undergo MT, neurological complications, particularly AIS, are common owing to the predominance of left-atrium CM. However, it is worth noting that CM at other sites can result in the same phenomena. It is not entirely surprising that CMs located in the left ventricle or aortic valve can easily release emboli into the systemic circulation (17, 18). Indeed, MT-treated AIS secondary to left ventricle myxoma has been reported previously (17). A recent systematic review on aortic valve myxoma indicated that cerebrovascular events are one of the most common presentations of aortic valve myxoma (19). Interestingly, Rao et al. reported an extremely rare case of recurrent stroke that was the result of a right ventricle myxoma and a patent foramen ovale. The authors speculated that the paradoxical embolism of tumor tissue via the intracardiac shunt contributed to the recurrent ischemic episodes (20).

AIS is the most common initial manifestation that affected almost 80% of our cases, which is in line with previous reports (10, 11). Although intracardiac obstruction, non-specific constitutional symptoms, and peripheral embolism are common in CM patients (2–4), very few patients with MT-treated CM-AIS exhibited such symptoms. Moreover, even fewer patients exhibited clinical symptoms before the onset of AIS. This can result in CM being frequently misdiagnosed until the occurrence of AIS. Compared with previous reports, the significantly lower prevalence of systemic presentations in our cohort may be attributed to limitations of the study design, which include the retrospective nature and potential selection bias. Furthermore, minor and non-specific symptoms may be neglected in medical records. In our study, diagnosis of CM preceded AIS in six patients and was delayed until AIS onset in 29 patients. This result is consistent with a previous comprehensive literature review of 133 CM-related stroke patients (12). However, there were no significant differences in clinical outcomes between patients with pre-MT diagnosed CM and post-therapy diagnosed CM. Thus, the decision to administer reperfusion treatment should not be delayed by the work-up of CM patients.

The predominant neuroimaging patterns were scattered, with multiple phases of infarctions in multiple vascular territories owing to the embolic nature of CM-AIS. Emboli were preferentially lodged into the anterior circulation, whereas the MCA was the most affected branch. Previous studies have shown that cardiogenic embolisms have a rightward propensity for cerebral infarcts because of the anatomical structures and the rheological properties of the aortic arch (21). A recent study of infarct lesion mapping analysis in a Swiss-AF cohort also revealed that large emboli preferentially enter the right hemisphere of the brain. The right common carotid artery is the first branch and the largest caliber of the aortic arch. Its angulation to the aortic arch is less than other branches of the aortic arch, which consequently increases the probability of emboli entering the right hemisphere (22). Patients with large right hemispheric infarctions have been shown to be more likely to have cardioembolism stroke and atrial fibrillation than patients with left-hemispheric infarctions (23). Thus, theoretically, CM-AIS may also have a similar rightward propensity. However, the opposite has been reported, whereby the left-hemispheric territory is dominant in CM-AIS patients, regardless of whether they have undergone reperfusion treatment (12, 14, 16). A possible interpretation for this phenomenon is that CM emboli are different from other emboli. Another interpretation is that patients selected to undergo IVT and MT have more severe clinical manifestations, which are usually caused by left-hemispheric territory involvement. The absence of clinical symptoms in patients with right hemisphere lesions is not unusual, and this may also contribute to the discrepancy. In contrast, the hyperdense artery sign is common in LVO patients, with a reported prevalence of up to 55.7% (24). However, it is uncommon in MT-treated CM-AIS patients. Moreover, one patient was reported to have a hypodense artery sign due to the presence of a fatty embolus, which was later identified as a component of CM (25).

Regarding thrombolytic therapy in CM-AIS patients, although it remains the first-line treatment for AIS, its safety and efficacy have not been established in CM-AIS patients. Because of the high theoretical risk of ICH as a result of CM-related cerebral aneurysms, CM was initially considered a contraindication to thrombolytic therapy. The reported prevalence of myxomatous aneurysms in CM patients varies from 0 to 67% across different studies (10, 13, 26, 27). However, the available epidemiological data are derived from small case series, which clouds the actual incidence of myxomatous aneurysms. The pathophysiological mechanism underlying the development of myxomatous aneurysms remains unclear, although the neoplastic process and micro-embolic damage theories are the most commonly accepted theories (28). Myxomatous emboli penetrate the endothelium of the vessel wall, damage the architecture of the vessel, proliferate within the subintimal layer, initiate inflammatory cascades, and eventually form an aneurysm (28). A recent systematic review of 41 cases with CM-related aneurysms reported that most aneurysms are located on the MCA (92.7%), followed by the ACA (65.9%) and PCA (34.1%). One-quarter of patients had a cerebral hemorrhage due to aneurysm rupture (29). The rupture rate of CM-related aneurysms is higher than that of general intracranial aneurysms (1–3% annually) (29). However, the majority (68.3%) of cases were managed conservatively, and most had no hemorrhage complications (1/28 patients, 3.6%) or aneurysm enlargement (3/28 patients, 10.7%) during an average follow-up duration of 40 months (29). A recent study on 412 AIS patients with unruptured intracranial aneurysms demonstrated that thrombolytic therapy does not increase the risk of ICH or sICH in this cohort (30). However, because of the varied pathological mechanisms between myxomatous and intracranial aneurysms, these results cannot be directly applied to clinical decision-making for CM-AIS patients. A recent comprehensive literature review of thrombolytic therapy for CM-related stroke described the safety and efficacy features in 22 cases with this rare condition (6). The reported prevalence of ICH was 22.7%. Of these patients who experienced hemorrhagic complications, no major clinical deterioration or death was observed following hemorrhage. The reported prevalence of ICH was similar to that reported in the SITS-MOST (14.5%) and ECASS III studies (27%). For the efficacy of IVT, they found that 81.8% of cases showed clinical improvements. Thus, the authors suggested that IVT is safe and effective for patients with CM-AIS (6). However, none of the patients in our study showed clinical improvement following IVT. Reasons for this discrepancy may include differences in the composition of the embolus, the presence of an LVO, and the selection and publication biases. An embolus in patients with CM-AIS may be part of the CM, a thrombus on the surface of the CM, or a mixture of both (12). IVT may only be effective for breaking down the components of the thrombus. In our patients, most of the retrieved emboli were myxomatous tissue, which may contribute to the failure of IVT. However, there was no significant difference in the prevalence of HT between patients who underwent IVT and those who did not undergo IVT. Therefore, IVT should not be withheld when CM-AIS is highly suspected. Only when AIS secondary to infective endocarditis cannot be excluded should IVT be critically discussed, given its significantly higher risk of sICH (31). In addition, cerebral angiography using CTA or MRA is recommended to be performed prior to IVT for cerebral vessel evaluation, bleeding risk stratification, and minimizing the risk of ICH (6, 10).

MT is currently the established treatment for AIS with LVO. However, there is limited evidence on the safety and efficacy of MT in CM-AIS patients. Our case series is the largest case series conducted in these patients to date, and almost all cases from the literature were included in our systematic review. In our study, the occurrence of a good outcome and sICH (60 and 5.7%, respectively) was similar to those reported in the MR CLEAN (32.6 and 7.7%, respectively) and REVASCAT studies (43.7 and 1.9%, respectively) (32). In addition, MT allows for the valuable histological analysis of the retrieved embolus, which can offer clues regarding the pathogenesis of AIS. Therefore, MT may be considered a better early option for CM-AIS patients with LVO or add-on therapy for IVT. The most common approaches of MT are SR and DA techniques. SR techniques are the most commonly utilized techniques and the primary treatment used in most randomized trials on endovascular therapy for AIS (33). The 2019 American Heart Association/American Stroke Association (AHA/ASA) guideline for managing AIS states that SR techniques remain the first-line approach for MT (34). Recent evidence indicates that DA techniques are effective and safe alternatives to SR techniques with a similar rate of favorable function outcomes and mortality (35). In clinical practice, the choice of the MT method depends on the characteristics of the clot. The size, composition, fragility of the embolus, and the anatomy of the occluded artery play critical roles in the degree of successful recanalization and the occurrence of new embolic events (36). However, because of the inadequate sample size of our study, we did not observe significant differences among the various techniques and devices. Our systematic review revealed that DA tended to provide more first-attempt successful reperfusions and favorable function outcomes but fewer distal embolism and hemorrhagic complications than SR in patients with CM-AIS. Usually, the embolus of CM-AIS patients is gelatinous, fragile, and flexible. Moreover, they may be fragmented with the expansion and withdrawal of the SR and likely require more thrombectomy attempts, which may increase the risk of ICH. Depending on the different mechanisms of action of SR and DA, SR may be more harmful to the vascular endothelium. Bourcier and colleagues found an increased risk of ICH and poor outcome in patients who required more than three thrombectomy attempts in the SR group than in the DA group (18). In our study, eight out of nine cases (the thrombectomy technique was not described in one case) who developed hemorrhagic complications had undergone the SR procedure, and five had received more than three passes. In addition, the fragile and flexible nature of emboli from a CM is likely to cause emboli migration when using SR. In contrast, emboli are often readily retrieved using DA. Therefore, aspiration thrombectomy may be a reasonable choice as first-line MT in CM-AIS patients. However, our case series found the opposite, which may be because the widely used Penumbra System was rarely used in our study because they are not covered by medical insurance in our province. Thus, our neurointerventionalists used other catheters with manual aspirations, which may have contributed to the low efficacy of DA. Further studies in a larger cohort may help determine the superiority of MT techniques for treating this rare condition.

Regarding the diagnostic strategies for CM, a timely and precise diagnosis is crucial. Cardiac imaging should be undertaken as soon as possible. Most CMs can be easily detected using echocardiography, and TTE is the primary screening tool for the diagnosis of CMs. However, it is worth noting that TTE failed to detect cardiac abnormalities in several patients. In contrast, TEE has higher diagnostic accuracy and provides a better morphologic definition of the characteristics, location, and attachment point of CM (37). In cases where a diagnosis is still not definite, cardiac MR can offer valuable information to help characterize CM. Although the use of chest CT scans for CM diagnosis is limited, it may be an alternative second-line diagnosis strategy that is particularly useful for detecting calcifications and hemorrhages (38).

In terms of surgical resection treatment for CM-AIS patients, recent evidence recommends that surgical resection of the CM should be performed as early as possible if clinical condition allows. There are several reasons for performing surgical resection early. First, CM heightens the risk of embolism, metastasis, aneurysm, intracardiac obstruction, and even sudden death. A previous comprehensive review described that patients who did not undergo CM resection had significantly higher mortality (44.4%) than those who underwent CM resection (2.1%) (12). Second, pharmacological treatment, such as antiplatelet and anticoagulation agents, provides inadequate therapeutic benefit for preventing embolism events (12). For example, one study reported that cerebral embolism events occurred in almost half of all CM patients who were under pharmacological treatment (10). Additionally, CVE recurrence was observed in 23% of patients who received bridging-antithrombotic treatment during the interval between CVE onset and CM surgery (10). In line with these findings, two of our patients experienced stroke recurrence during the bridging interval. Third, no operation-related severe complications or deaths were observed in our case series of previous reports. The operative mortality rate of CM resection is reported to be as low as 0–3% (39). Moreover, there is no difference in mortality between patients who undergo early resection and delayed resection (12). In contrast, the risk of cerebral embolism gradually increases with the elapsed time between the AIS and CM surgical resection (10). Complications are rare for CM resection, although sick sinus syndrome, atrial fibrillation, and deep wound infection have been occasionally reported (2, 4, 10). In addition, no HT was observed in patients who underwent early resection (10). Taken together, the above evidence suggests that surgical resection is imperative, feasible, and safe for the treatment of CM.

### Limitations

The present study has several limitations, the most notable of which are related to the small sample size, the retrospective nature of the study, and the selection of reported cases. Despite our collaborative effort of multiple centers to assemble a larger case series of CM-AIS patients treated by MT, patient numbers in our case series remained modest because of the rarity of the disease. As a result, this led to low overall power and reliability of the study and prevented the direct application of our results to improving clinical practice guidelines. Additionally, the variable quality of the included publications may have influenced the results. The clinical data, which included radiological data, procedural characteristics, and myxoma and embolus characteristics, were incomplete in several publications. Furthermore, our study had several biases, which included the heterogeneity of publications, publication bias, selection bias, and citation bias. Nevertheless, the rarity of CM-AIS presents challenges for conducting prospective studies and clinical randomized controlled trials, especially those investigating CM-AIS patients who undergo MT treatment. Thus, a systematic review of case reports and case series for rare diseases is worthwhile.

## Conclusion

Based on our case series and pooled analysis of the available literature, we revealed that the use of MT in CM-AIS patients with LVO remains uncommon; however, MT has a high recanalization rate and low bleeding risk and allows the exploration of stroke etiology via histopathological analysis of retrieved emboli. Thus, MT appears to be a feasible, effective, and potentially beneficial therapeutic option for CM-AIS patients. Further research is needed to gather additional evidence on the efficacy, safety, and strategies of MT in this particular population.

## Data Availability Statement

The original contributions presented in the study are included in the article/Supplementary Material, further inquiries can be directed to the corresponding author/s.

## Ethics Statement

The studies involving human participants were reviewed and approved by the Ethics Committee of the Second Affiliated Hospital of Wenzhou Medical University. The patients/participants provided their written informed consent to participate in this study. Written informed consent was obtained from the individual(s) for the publication of any potentially identifiable images or data included in this article.

## Author Contributions

FF and XC contributed to the design of the article and are responsible for the integrity and accuracy of the data. JR and ZT contributed to the acquisition and analysis of the clinical data, drafting the manuscript, and reviewing the published literature. QB, MX, MJ, and XW contributed to clinical data collection and follow-up. YL, BY, and DL contributed to the neurointerventional data collection and analysis. All authors read and approved the final manuscript to be published.

## Funding

This work was supported by the Medical Science and Technology Project of Zhejiang Province of China (No.2021KY797 and No.2022KY454) and the Wenzhou Basic Scientific Research Project (No. Y20210900).

## Conflict of Interest

The authors declare that the research was conducted in the absence of any commercial or financial relationships that could be construed as a potential conflict of interest.

## Publisher's Note

All claims expressed in this article are solely those of the authors and do not necessarily represent those of their affiliated organizations, or those of the publisher, the editors and the reviewers. Any product that may be evaluated in this article, or claim that may be made by its manufacturer, is not guaranteed or endorsed by the publisher.
