# Supplementary material for: Mechanical Thrombectomy for Acute Ischemic Stroke in Patients With Cardiac Myxoma: A Case Series and Pooled Analysis
Source: Front Neurol. 2022 Apr 18;13:877056. doi: 10.3389/fneur.2022.877056 (PMC9058073; doi:10.3389/fneur.2022.877056)
Supplement: Supplementary file 1 [file Table_1.docx]

Supplementary Table 1: Result of the systematic literature review of reports on mechanical thrombectomy for CM-AIS patients

| **Study**  **(year)** | **Age/Sex** | **Risk**  **Factor** | **Initial**  **NIHSS** | **HDA** | **Multi-**  **vessel**  **Involved** | **IVT**  **(effect)** | **OTN**  **(min)** | **Other**  **vascular**  **abnormlity** | **Mechanical Thrombectomy** | | | | | **HT** | **sICH** | **Cardiac Myxoma** | | | | **CVE**  **relapse**  **before**  **removal** | | **mRS at follow-up** |  |
| --- | --- | --- | --- | --- | --- | --- | --- | --- | --- | --- | --- | --- | --- | --- | --- | --- | --- | --- | --- | --- | --- | --- | --- |
|  |  |  |  |  |  |  |  |  | **Occlusion**  **Site** | **Device, Type, Passage, Recanalization** | **TICI** | **Embolus (appearance, histology)** | **NIHSS** |  |  | **Diagnostic**  **Method** | **Site and Diameter** | **Pre-stroke**  **Symptoms** | **Removal**  **Time**  **(day)** |  |  |  |  |
| Garcia-Ptacek, 2014  (1) | 45/NA | No | 22 | No | Yes | Yes  (Worse) | 100 | Bilateral iliac and distal aortic thrombosis | Left M1 | Solitaire FR 4×20, SR, 4, failed  Trevo, SR, 1, failed;  Wingspan 3×20, stent, 1, failed  PHAROS Vitesse, stent, 1, failed | 0 | NA, myxoma | 31 | No | No | TTE | Left atrium, NA | No | NA | No | | 4 |  |
|  | 34/NA | No | 26 | No | Yes | Yes  (NE) | 190 | No | Left M1  Left A2 | Solitaire FR 4×20, SR, 1, pass  Solitaire FR 4×20, SR, 1, pass | 3  3 | Tumorous embolus, myxoma | NA | No | No | TTE | Left atrium, NA | No | NA | No | | 2 |  |
| Kamiya, 2014  (2) | 48/F | No | 32 | No | Yes | Yes  (NE) | 120 | No | BA  Right M2 | Spontaneous recanalization  Intra-arterial thrombolysis, 2, failed, then Soutenir retrieval device, NA, failed | 3  0 | NA | NA | No | No | TTE | Left atrium,  54 mm | No | 20 | No | | 5 |  |
| Baek, 2014  (3) | 46/M | No | NA | No | No | Yes  (NE) | NA | Cilioretinal artery | Left ICA  Left M2 | Optimo 9-F balloon-tipped guiding catheter, DA, 1, pass  Penumbra 032 system reperfusion catheter DA, 1, pass | 2b  3 | Yellowish and gelatinous tissue, myxoma | NA | No | No | TTE | Left atrium,  42 mm | No | 5 | No | | 1 |  |
| van den Wijngaard, 2014  (4) | 14/M | No | 21 | No | No | Yes  (NE) | 240 | Three small m3 aneurysms | Left M1 | Solitaire FR 4×20, SR, 1, pass | 2b | NA | NA | No | No | ECG | Left and right atrium,  NA | Typical skin lesions | 1 | Central  retinal artery occlusion on Day 2 | | 2 |  |
| Ryu, 2015  (5) | 34/M | Smoking | 9 | Yes | No | Yes, 0.6mg/kg  (NE) | 113 | No | Right M1 | Merci L5 Retriever, SR, 3, pass | 3 | Elastic white embolus, myxoma | 6 | Yes, NA | No | TEE | Left atrium,  14 mm | No | 1 month | No | | 2 |  |
| Vega, 2015  (6) | 11/M | No | 16 | No | Yes | No | NA | No | Right M1  Right M2  Right PCoA | Trevo Retriever, SR,3, failed, then Penumbra 041 System, DA, 1, pass  Penumbra System 026, DA, NA, pass  Penumbra System 026, DA, NA, pass | 2b  2b  3 | Fatty, gelatinous, and friable embolus, NA | 7 | No | No | TEE | Left atrium,  40 mm | Raynaud syndrome, recurrent rash, syncope | 2 | No | | 1 |  |
| Uneda, 2016  (7) | 70/M | No | 11 | NA | No | Yes  (NE) | 175 | No | Left T-ICA  Left M2 | Penumbra 5MAX ACE, DA, 3, pass  Trevo retriever, SR, 1, pass | 2b  2b | Whitish-to-reddish, gelatinous, and friable embolus, myxoma | NA | No | No | TEE | Left atrium,  40 mm | No | 6 | No | | 2 |  |
| Zander, 2016  (8) | 46/F | No | 16 | No | No | No | NA | No | Right M1 | Solitaire AB Retriever, SR, 1, pass | 3 | Tissue fragment, myxoma | 6 | No | No | TEE | Left atrium,  115 mm | No | 6 | No | | 0 |  |
| McGowan, 2016  (9) | 21/F | No | 12 | No | Yes | No | NA | Aortic and the  superior mesenteric artery emboli | Left M1  Basilar artery | Trevo stent retriever, SR, 1, pass  Stent-retriever, SR, NA, in next hospital | 3  3 | Fatty appearing embolus with  no visible thrombus, NA | NA | No | No | Cotrast CT | Left atrium,  NA | No | 1 | No | | 0 |  |
| Chung, 2016  (10) | 4/M | No | 16 | No | No | YES, 0.6mg/kg  (NE) | 210 | No | Left M1 | Solitaire FR 4× 20, SR, 1, pass | 3 | Brown gelatinous material, myxoma | NA | No | No | TTE | Left atrium,  20 mm | Febrile  convulsion | 7 | No | | 1 |  |
| Zapata-Arriaza, 2017  (11) | 17/M | Yes | 32 | No | Yes | Yes  (NE) | 95 | Left subclavian artery, abdominal  Aorta, both iliac arteries, both femoral arteries | Right ICA  Rright M1  Left M1-M2 | Solitaire FR Retriever, SR, 2, pass  Solitaire FR Retriever, SR, 2, pass  Solitaire FR Retriever, SR, 2, pass | 3  3  3 | Fatty appearing embolus, myxoma | NA | Yes,  PH2 | Yes | TTE | Left atrium,  NA | No | No | No | | 6 |  |
| Kuwahara, 2018  (12) | 31/M | No | 9 | No | No | Yes  (NE) | 87 | No | Right M2 | Penumbra 5MAX ACE, DA, 1, pass | 3 | Myxoma-like embolus, myxoma | 0 | No | No | TTE | Left atrium,  NA | No | 6 | No | | 0 |  |
| Tadi, 2019  (13) | 23/F | Migraines  NSAIDs | 19 | No | No | Yes  (NE) | NA | No | Left M1 | NA | 3 | NA | 10 | No | No | TTE | Left atrium,  70 mm | No | 8 | New infarct on Day 7 | | 2 |  |
| Cai, 2019  (14) | 61/M | No | 16 | No | No | Yes  (NE) | NA | No | Left M1 | Navein, DA, 1, pass | 3 | Transparent and jelly embolus, myxoma | 5 | No | No | TTE | Left atrium,  45 mm | No | 21 | No | | 0 |  |
| Li, 2019  (15) | 61/M | No | 14 | No | Yes | Yes  (NE) | 2h | Left external carotid artery | Left M1 | 5F Navein, DA, 1, pass | 3 | Gelatinous thrombus, myxoma | NA | No | No | TTE | Left atrium,  32 mm | No | 30 | No | | 2 |  |
| Tona, 2020  (16) | 12/F | No | NA | No | No | No | NA | No | Left M1 | Retriever, SR, 1, pass | 2b | Yellowish  and jelly thrombus, NA | NA | No | No | TTE | Left atrium,  35 mm | No | 2 | No | | 3 |  |
| Zhou , 2020  (17) | 16/F | No | 20 | No | No | No | NA | No | Left M2 superior trunk  Left M2 inferior trunk | Trevo Retriever, SR, 1, pass  Trevo Retriever, SR, 3, failed | 3  2a | Gray-white, gelatinous and fragile tumor emboli, NA | NA | No | No | TTE | Left atrium,  48mm | No | 15 | No | | 1 |  |
| Mikulenka, 2020  (18) | 21/F | Migraines  Contracep-tives | 11 | No | Yes | No | NA | No | Left M1  Left ACA | NA | NA | NA | NA | No | No | TTE | Left ventricle, 23 mm | No | Resected | No | | 3 |  |
| Coffee, 2020  (19) | 12/F | Familial history of Carney complex | NA | No | Yes | No | NA | Abdominal aorta,bilateral iliac and popliteal arteries | Left M1 | NA | 3 | Yellow discoloration and gelatinous specimen, myxoma | NA | Yes, HI2 | No | TTE | Left atrium,  27 mm | No | 10 | No | | 3 |  |
| Zhang, 2020  (20) | 15/F | No | 26 | Yes | No | Yes  (NE) | 60 | No | Left M1 | Navien, DA, 1, failed  Solitaire AB, SR,1, pass | 3 | Gelatinous thrombus, myxoma | 26 | Yes, PH2 | No | TTE | Left atrium,  35 mm | No | Not performed | No | 3 | | |
| Bhatia, 2021  (21) | 42/F | No | 28 | No | No | No | NA | No | Distal BA | Trevo XP 4× 30, SR, 1, pass | 3 | NA | 10 | No | No | ECG | Left atrium,  25 mm | No | 1 month | No | 1 | | |
| Chang, 2021  (22) | 42/F | No | 18 | No | No | Yes  (NE) | 140 | No | Left M1 | Solitaire FR, SR, 1, pass | 3 | Red jelly-like thrombus, myxoma | 3 | No | No | TTE | Left atrium,  65 mm | No | 2 | No | | 0 | |
| Bedoya, 2021  (23) | 43/F | Smoking | 9 | No | No | No | NA | No | Right M1 | Unknown stent retriever, SR, 1, pass | 3 | NA | NA | Yes,  SAH | No | TTE | Left atrium,  80 mm | Chest pain, shortness of breath when walking | Resected | No | | 3 | |

ACA: anterior cerebral artery; BA: basilar artery; DA: direct aspiration; F: female; HDA: high-density sign of artery; HT: hemorrhagic transformation; ICA: internal carotid artery; IVT: Intravenous thrombolysis; M: male; MCA: middle cerebral artery; mRS: modified Rankin Scale; NA: not available; NE: not effective; NIHSS: National Institutes of Health Stroke Scale; PCA: posterior cerebral artery; PCoA: posterior communicating artery; PH2: parenchymal hematoma type II; SAH: subarachnoid hemorrhage; sICH: symptomatic intracranial hemorrhage; SR: stent retriever; TEE: transesophageal echocardiography; TICI: Thrombolysis in Cerebral Ischemia; TTE: transthoracic echocardiography; VA: vertebral artery.

Reference

1. Garcia-Ptacek S, Matias-Guiu JA, Valencia-Sanchez C, Gil A, Bernal-Becerra I, De las Heras-Revilla V, et al. Mechanical endovascular treatment of acute stroke due to cardiac myxoma. *J Neurointerv Surg* (2014) 6(1):e1. Epub 2012/07/14. doi: 10.1136/neurintsurg-2012-010343. PubMed PMID: 22791184.

2. Kamiya Y, Ichikawa H, Mizuma K, Itaya K, Shimizu Y, Kawamura M. [Case of acute ischemic stroke due to cardiac myxoma treated by intravenous thrombolysis and endovascular therapy]. *Rinsho Shinkeigaku* (2014) 54(6):502-6. Epub 2014/07/06. doi: 10.5692/clinicalneurol.54.502. PubMed PMID: 24990835.

3. Baek SH, Park S, Lee NJ, Kang Y, Cho KH. Effective mechanical thrombectomy in a patient with hyperacute ischemic stroke associated with cardiac myxoma. *J Stroke Cerebrovasc Dis* (2014) 23(9):e417-9. Epub 2014/09/02. doi: 10.1016/j.jstrokecerebrovasdis.2014.05.006. PubMed PMID: 25174564.

4. van den Wijngaard I, Wermer M, van Walderveen M, Wiendels N, Peeters-Scholte C, Lycklama ANG. Intra-arterial treatment in a child with embolic stroke due to atrial myxoma. *Interv Neuroradiol* (2014) 20(3):345-51. Epub 2014/07/01. doi: 10.15274/NAJ-2014-10026

10.15274/INA-2014-10026. PubMed PMID: 24976098; PubMed Central PMCID: PMCPMC4178761.

5. Ryu B, Ishikawa T, Sato S, Yokote A, Nakamoto H, Nie M, et al. Mechanical Endovascular Recanalization in a Patient with Middle Cerebral Artery Occlusion by Tumorous Emboli Originating from Cardiac Myxoma. *NMC Case Rep J* (2015) 2(2):53-6. Epub 2015/03/23. doi: 10.2176/nmccrj.2014-0359. PubMed PMID: 28663964; PubMed Central PMCID: PMCPMC5364909.

6. Vega RA, Chan JL, Anene-Maidoh TI, Grimes MM, Reavey-Cantwell JF. Mechanical thrombectomy for pediatric stroke arising from an atrial myxoma: case report. *J Neurosurg Pediatr* (2015) 15(3):301-5. Epub 2015/01/07. doi: 10.3171/2014.10.PEDS14292. PubMed PMID: 25559920.

7. Uneda A, Suzuki K, Hirashita K, Yoshino K. Tandem cervical/intracranial internal carotid artery occlusion due to cardiac myxoma treated successfully with mechanical endovascular thrombectomy. *Acta Neurochir (Wien)* (2016) 158(7):1393-5. Epub 2016/05/25. doi: 10.1007/s00701-016-2833-1. PubMed PMID: 27216756.

8. Zander T, Maynar J, Lopez-Zarraga F, Herrera R, Timiraos-Fernandez JJ, Saraceni A, et al. Mechanical thrombectomy in patients with tumour-related ischaemic stroke. *Interv Neuroradiol* (2016) 22(6):705-8. Epub 2016/09/30. doi: 10.1177/1591019916669853. PubMed PMID: 27683226; PubMed Central PMCID: PMCPMC5564369.

9. McGowan AR, Thibodeau C, McGowan A. Intracranial and visceral arterial embolization of a cardiac myxoma that was treated with endovascular stent-retriever therapy. *Interv Neuroradiol* (2016) 22(5):535-9. Epub 2016/06/17. doi: 10.1177/1591019916653939. PubMed PMID: 27306523; PubMed Central PMCID: PMCPMC5072215.

10. Chung YS, Lee WJ, Hong J, Byun JS, Kim JK, Chae SA. Mechanical thrombectomy in cardiac myxoma stroke: a case report and review of the literature. *Acta Neurochir (Wien)* (2016) 158(6):1083-8. Epub 2016/04/05. doi: 10.1007/s00701-016-2780-x. PubMed PMID: 27040551.

11. Zapata-Arriaza E, Pardo-Galiana B, González-García A, Montaner Villalonga J. Intravenous thrombolysis and thrombectomy in young patients with ischaemic stroke due to undetected atrial myxoma: Do recent clinical trials provide sufficient evidence to support reperfusion in these cases? *Neurología (English Edition)* (2017) 32(6):404-7. doi: 10.1016/j.NAleng.2016.11.001.

12. Kuwahara K, Moriya S, Maeda S, Hayakawa M, Mizoguchi Y, Nakahara I, et al. Cardiogenic Embolism due to Left Atrial Myxoma Successfully Treated by ADAPT: A Case Report. *Journal of Neuroendovascular Therapy* (2018) 12(4):193-8. doi: 10.5797/jnet.cr.2017-0070.

13. Tadi P, Feroze R, Reddy P, Sravanthi P, Fakhri N, McTaggart R, et al. Clinical Reasoning: Mechanical thrombectomy for acute ischemic stroke in the setting of atrial myxoma. *Neurology* (2019) 93(16):e1572-e6. Epub 2019/10/16. doi: 10.1212/WNL.0000000000008321. PubMed PMID: 31611322.

14. Cai X, Xu H, Liu J, Dai Y, He W, Li J, et al. Endovascular thrombectomy after intravenous recombinant tissue plasminogen activator (bridging therapy) for embolic stroke due to cardiac myxoma: a case report. *Chinese Journal of Neurology* (2020) 53(2):118-21.

15. Li J, Tang X, Cai X, Wang K, Yan J. Mechanical thrombectomy for middle cerebral artery embolization caused by atrial myxoma: a case report and literature revie. *Chinese Interventional Neurology Meeting 2019-The 15th International Stroke Summit* (2019):88-9.

16. Tona C, Nosadini M, Pelizza MF, Pin JN, Baggio L, Boniver C, et al. Cardiac Myxoma as a Rare Cause of Pediatric Arterial Ischemic Stroke: Case Report and Literature Review. *Neuropediatrics* (2020) 51(6):389-96. Epub 2020/05/06. doi: 10.1055/s-0040-1710338. PubMed PMID: 32369836.

17. Zhou B, Huang S, Liu S, Ren L, Huang C, Lian Z. Thrombectomy for Stroke Caused by Cardiac Myxoma. *J Stroke Cerebrovasc Dis* (2020) 29(12):105407. Epub 2020/12/02. doi: 10.1016/j.jstrokecerebrovasdis.2020.105407. PubMed PMID: 33254380.

18. Mikulenka P, Stetkarova I, Vasko P, Peisker T. Left ventricle cardiac myxoma as a cause of ischaemic stroke in young patient treated by mechanical thrombectomy. *Neuro Endocrinol Lett* (2020) 41(3):109-12. Epub 2020/11/18. PubMed PMID: 33201646.

19. Coffee E, Sankhla N, Bass R, Dure L, Rashid S. Child Neurology: Arterial ischemic stroke in a 12-year-old patient with cardiac myxomas. *Neurology* (2020) 94(10):e1103-e6. Epub 2020/02/20. doi: 10.1212/WNL.0000000000009060. PubMed PMID: 32071165.

20. Zhang P, Hu F, Sun Y, Niu W, Wang J, Guo W. One case report of intravenous thrombolysis bridging endovascular thrombectomy for ischemic stroke caused by juvenile atrial myxoma. *Chinese Journal of Nervous and Mental Diseases* (2020) 3:166-8.

21. Bhatia V, Jain C, Ray S, Gupta O, Chatterjee D, Kumar A. Mechanical Thrombectomy in Embolic Cardiac Myxoma: Case Report and Literature Review. *Neurol India* (2021) 69(3):707-10. Epub 2021/06/26. doi: 10.4103/0028-3886.319216. PubMed PMID: 34169873.

22. Chang WS, Li N, Liu H, Yin JJ, Zhang HQ. Thrombolysis and embolectomy in treatment of acute stroke as a bridge to open-heart resection of giant cardiac myxoma: A case report. *World J Clin Cases* (2021) 9(25):7572-8. Epub 2021/10/08. doi: 10.12998/wjcc.v9.i25.7572. PubMed PMID: 34616828; PubMed Central PMCID: PMCPMC8464453.

23. Bedoya RA, Smith T, Ma H, Goodner A, Sreedhar J. Incidental Finding of an Exceptionally Large Left Atrial Myxoma Presenting as an Acute Cardioembolic Stroke. *Cureus* (2021) 13(9):e18056. Epub 2021/10/26. doi: 10.7759/cureus.18056. PubMed PMID: 34692285; PubMed Central PMCID: PMCPMC8523365.
